# Endometriosis within the sigmoid colon: A rare cause of bowel obstruction

**DOI:** 10.12669/pjms.36.6.2525

**Published:** 2020

**Authors:** Zheng Long-zhi, Guo Jian, Lin Wei

**Affiliations:** 1 Dr. Zheng Long-zhi, PhD. Associate Chief Physician, Department of Gastrointestinal Surgery, The Affiliated Hospital of Putian University, Putian, Fujian Province, People’s Republic of China; 2 Dr. Guo Jian, Attending Doctor, Department of Gastrointestinal Surgery, The Affiliated Hospital of Putian University, Putian, Fujian Province, People’s Republic of China; 3 Dr. Lin Wei, PhD. Chief Physician, Department of Gastrointestinal Surgery, The Affiliated Hospital of Putian University, Putian, Fujian Province, People’s Republic of China

**Keywords:** Bowel endometriosis, Sigmoid colon, Diagnosis and treatment

## Abstract

Endometriosis is a common gynecological disease, ectopic endometrium can invade any part of the body, usually in the ovary and uterine sacral ligament, while endometriosis invades the intestinal wall to cause intestinal obstruction is very rare, which easily leads to misdiagnosis. In this case report, we present a case of sigmoid endometriosis with bowel obstruction. Pathological examination is the main basis for the diagnosis of intestinal endometriosis, and the comprehensive treatment of surgery and hormonal therapy is an effective method for the treatment of intestinal endometriosis.

## INTRODUCTION

Endometriosis is a frequent and common disease in women of childbearing age. It refers to a type of chronic inflammatory reaction caused by the presence of active endometrium tissue (glands and stroma) outside the uterus. Endometriosis often occurs in the reproductive organs and is most common in the ovaries. It affects about 10% of women of childbearing age, and about 35~50% of these patients have pelvic pain and infertility.^1^ Intestinal endometriosis, a component of endometriosis, is relatively rare in clinical practice, accounting for about 3.8%~37% of patients with endometriosis.^2^ It is most common in rectum and sigmoid colon, followed by appendix and terminal ileum, and rare in other intestines. The clinical manifestations of intestinal endometriosis are mostly atypical, and the cases of intestinal obstruction caused by it are even more rare in clinical practice, so early diagnosis is difficult and the rate of misdiagnosis is relatively high. Therefore, the knowledge related to intestinal endometriosis still deserves the attention of clinicians. In this paper, a case of intestinal endometriosis complicated with intestinal obstruction in our hospital was retrospectively analyzed, and relevant literatures were reviewed to explore the effective means of examination and clinical treatment of intestinal endometriosis.

## CASE REPORT

**Fig.1:**
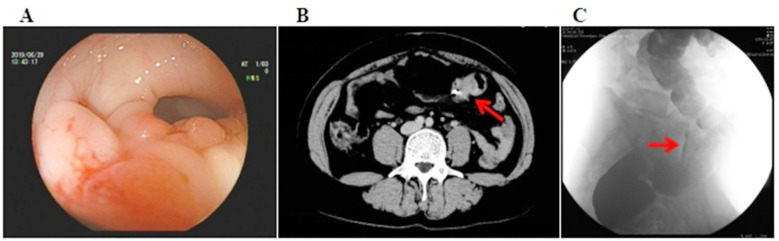
A, Colonoscopy showed an irregularly protrude lesion in the sigmoid colon about 19cm from the anal verge. The surface was rough and granular, invading about 1/2 of the bowel, and the bowel lumen was stiff with stenosis. B, Pelvic CT scan showed local thickening of the intestinal wall in the left lower abdomen, and the metal artifact was a titanium clip (which was clipped during the colonoscopy and can be x-rayed before surgery to determine the location of the mass). C, Barium enema showed mucosal abnormality and focal stenosis of the sigmoid region. The rest of the colon was normal.

A 43-year-old female patient was admitted to the hospital on July 10, 2019 due to “changes in bowel habits for more than one year”. The patient has been suffering from constipation for more than a year without obvious causes, accompanied by stool thinning and occasional mucosanguineous feces, which is not obviously related to the menstrual cycle. The patient considered “colitis” and can be relieved by taking oral drugs such as “Changyanning, Tablet, Po Chai Pills ” by herself, without going to the hospital for further examination and treatment. The patient had no previous history of abdominal surgery. Preoperative colonoscopy showed an irregularly protrude lesion in the sigmoid colon about 19cm from the anal verge. Colonoscopy biopsy showed a chronic inflammation of the sigmoid mucous membrane. Barium enema: mucosal abnormality and focal stenosis of the sigmoid region. Pelvic CT scan showed local intestinal wall thickening in the left abdomen (Fig.1).

**Fig.2:**
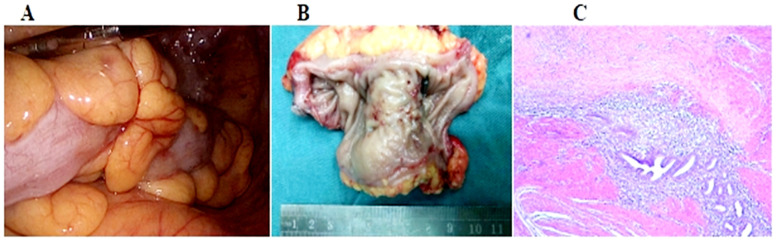
A, Intraoperative laparoscopic exploration revealed a hard mass in the sigmoid colon. B, Gross surgical pathology showed a sigmoid colon mass of approximately 3.0 cm x 4.0 cm, invading approximately one-half of the circumference. C, Histopathology showed endometrial glands and stroma in the tissues, and bowel endometriosis was considered.

Written informed consent was obtained from the patient for publication of this case report and any accompanying images.and the patient also agreed to undergo surgical treatment due to lumen stenosis and persistent symptoms. Intraoperative exploration showed no abnormalities in the liver, normal size and shape of uterus, normal ovary and fallopian tubes. Hard mass was palpable in sigmoid colon about 20cm from the anal verge, with a size of about 3.0cm × 4.0cm. The tumor was completely resected 2cm from the upper and lower edges of the tumor, and the pelvic cavity was thoroughly flushed. Postoperative pathology showed: sigmoid endometriosis (Fig.2). The patient recovered successfully and was discharged 10 days after surgery and continued hormone therapy with gonadotropin-releasing hormone analogues (GnRH-a). The patient was followed up well six months after the operation, and no symptoms such as abdominal distension, constipation and bloody stool occurred again.

## DISCUSSION

At present, preoperative diagnosis of intestinal endometriosis remains difficult, and some cases cannot be confirmed even by enteroscopy. In this case, colon cancer was considered at the first impression during colonoscopy, but was not supported by pathology. The pathology report was only ulcers with chronic inflammation, and the patient did not show symptoms of gastrointestinal tumors such as wasting and anemia during the onset. Since then, we considered that the clinical symptoms of the patients were not consistent with those of colorectal cancer, and the patient was relatively young, so a multidisciplinary consultation was organized. The possibility of intestinal endometriosis was considered after a multidisciplinary consultation with gastrointestinal surgeons, gynaecologists, imaging physicians and pathologists, combined with immunohistochemistry. Therefore, in clinical practice, women of childbearing age who have recurrent abdominal pain, abdominal distension, diarrhea, hematostool, intestinal obstruction, or who have the above symptoms after previous pelvic gynecological surgery, should have full detailed inquiry, considering the possibility of intestinal endometriosis.

The clinical manifestations of intestinal endometriosis vary according to the location and degree of intestinal involvement. The majority of the early lesions are small lesions located in the serosal surface or deep muscular layer, and the clinical symptoms are not obvious, only causing some non-specific symptoms, such as colic, constipation, etc. The large nodule lesions obviously involved in the intestinal wall may present pain and gastrointestinal symptoms of varying degrees, including abdominal pain, diarrhea, constipation, and abdominal distension. In severe cases, lumen stenosis caused by mass formation can lead to intestinal obstruction, which only accounts for 0.1% ~ 0.7%,^2^ and hemafecia is rare because lesions rarely invade the mucosa.

Colonoscopy, rectal endoscopic ultrasonography, transvaginal ultrasound (TVUS), CT, MRI, double-contrast barium enema is helpful for the diagnosis of intestinal endometriosis. While laparoscopy is still the gold standard for the identification and classification of endometriosis.^3^

The treatment principle of endometriosis is to eliminate and wipe out the lesion, alleviate and eliminate pain, improve and promote fertility, reduce and avoid recurrence. Once intestinal endometriosis is diagnosed, the patient must be counselled, and there are theoretically three options: expectant management, medical treatment, and surgery. So far, the effects of medical treatment on intestinal endometriosis have not been specifically studied, but hormone therapy for endometriosis is generally believed to not only inhibit ovulation, but may also be ineffective in cases of luminal stenosis is greater than 60%. And even if it does, intestinal symptoms will persist or recur when medication is discontinued.^4^ Therefore, surgery is still the main treatment for those who have obvious symptoms of intestinal endometriosis. There are three main surgical methods for patients with intestinal endometriosis: shaving excision, full thickness disc resection, and segmental excision and re-anastomosis. For patients without luminal stenosis, resection of the intestinal segment is not recommended, and removal of the lesion is acceptable. The surgical methods are usually shaving excision and full thickness disc resection. For patients with large lesions that cause luminal stenosis or even intestinal obstruction or recurrent hematoea, segmental excision and re-anastomosis is feasible. This surgical approach can completely remove the lesion, not only it can significantly improve gastrointestinal symptoms and quality of life,^5^ reduce the recurrence rate of endometriosis and increase fertility,^6^ but also can prevent the possibility of cancer.^7^ Postoperative follow-up plans (including whether to use hormone therapy after surgery) should also be developed in coordination with the department of gynaecology, as studies have shown that postoperative hormone therapy can prolong the interval between surgery and the first recurrence.^8^

In general, because intestinal endometriosis does not have typical clinical features and signs, preoperative diagnosis is difficult, so when women of childbearing age have intestinal obstruction and no other obvious reasons, endometriosis should be highly suspected. For intestinal endometriosis with stenosis or obstruction, surgical treatment is recommended, and laparoscopic surgery is preferred. In addition, the gastrointestinal surgeons should refer to the guidelines for diagnosis and treatment of endometriosis, and coordinate with the gynecologist to formulate the best treatment and follow-up plan for patients.

### Authors’ Contribution:

**LW:** Consultant surgeon who did surgery.

**ZLZ:** Study concept and analysis paper writing.

**GJ:** Literature review and assisted in surgery..
